# Effects of Fermentation and Oxidative Degradation on the Composition, Antioxidant Activity, ACE Inhibitory Activity, and In Vitro Neuroprotective Potential of Soybean-Derived Kefir Polysaccharide-Rich Extracts

**DOI:** 10.3390/foods15132372

**Published:** 2026-07-03

**Authors:** Wei-Cheng Hsiao, Taiki Miyazawa, Sue-Joan Chang, Yong-Han Hong, Yu-Chen Zhou, Man-Chu Deng, Teruo Miyazawa, Chun-Yung Huang

**Affiliations:** 1 Division of Gastroenterology, Department of Internal Medicine, Yuan’s General Hospital, Kaohsiung City 80249, Taiwan; 2 New Industry Creation Hatchery Center (NICHe), Tohoku University, Sendai 980-8579, Miyagi, Japan; 3 Department of Life Sciences, College of Bioscience and Biotechnology, National Cheng Kung University, Tainan 70101, Taiwan; 4 Marine Biology and Cetacean Research Center, National Cheng Kung University, Tainan 70101, Taiwan; 5 Graduate Program of Nutrition Science, National Taiwan Normal University (Gongguan Campus), Taipei City 11677, Taiwan; 6 Department of Seafood Science, National Kaohsiung University of Science and Technology, No. 142, Haijhuan Rd., Nanzih District, Kaohsiung City 81157, Taiwan

**Keywords:** bioactive polysaccharides, oxidative stress modulation, SH-SY5Y cell model, multivariate statistical analysis, heatmap clustering

## Abstract

Kefir is a probiotic beverage produced by symbiotic bacteria and yeasts. Polysaccharide-rich extracts from yellow and black soybeans (S and B) were obtained and subsequently fermented to produce S-F and B-F. The fermented extracts were further subjected to oxidative degradation using ascorbic acid and hydrogen peroxide to generate S-FD and B-FD. Physicochemical analyses revealed distinct differences in composition, phenolic profiles, and molecular weight among S-F, S-FD, B-F, and B-FD. Fourier transform infrared (FTIR) spectra indicated that oxidative degradation altered specific functional group intensities without disrupting the fundamental polysaccharide framework. Fermentation enhanced angiotensin-converting enzyme (ACE) inhibitory activity, and subsequent oxidative degradation further improved this effect. Both fermented and degraded extracts exhibited antioxidant activities, including 2,2-diphenyl-1-picrylhydrazyl (DPPH) and 2,2′-azino-bis(3-ethylbenzothiazoline-6-sulfonic acid) (ABTS) radical scavenging capacity, ferrous-ion chelating ability, and reducing power, with degraded samples showing greater activity. The effects of the extracts on SH-SY5Y human neuroblastoma cells were evaluated in vitro. No cytotoxicity was observed at concentrations up to 400 μg/mL. Treatment at 200 μg/mL increased cell viability and reduced apoptosis in rotenone (ROT)-treated cells. Multivariate analysis further indicated that oxidative degradation enhanced antioxidant and ACE inhibitory activities but may reduce the protective effects observed in SH-SY5Y cells. Overall, soybean-derived kefir polysaccharide-rich extracts show potential as functional ingredients for applications related to blood pressure regulation and antioxidant activity, while their protective effects in neuronal cell models warrant further investigation.

## 1. Introduction

Kefir is a traditional fermented dairy beverage originating from the Caucasus region and is increasingly recognized as a functional food because of its complex microbial ecology and diverse bioactivities. Unlike conventional fermented products produced using defined starter cultures, kefir is generated through the metabolic interactions of a symbiotic consortium of lactic acid bacteria, acetic acid bacteria, and yeasts embedded within a polysaccharide-protein matrix known as kefir grains. This dynamic microbial system produces various metabolites, including organic acids, ethanol, carbon dioxide, exopolysaccharides, and bioactive peptides, which contribute to its unique physicochemical and functional properties [1]. Recent studies have shown that the bioactivities of kefir are closely associated with its compositional complexity. Kefir-derived exopolysaccharides may exhibit antioxidant, immunomodulatory, and antimicrobial activities and may contribute to the regulation of gut microbiota and intestinal barrier function, highlighting their potential as health-promoting bioactive ingredients [2]. With increasing interest in food-derived polysaccharides, advanced purification and structural characterization approaches have become essential for understanding structure-function relationships and improving the development of functional polysaccharide-based products. Chromatographic purification, molecular weight analysis, monosaccharide profiling, and structural characterization are widely recognized as essential approaches for elucidating the biological activities of natural polysaccharides and supporting their applications in food and nutraceutical systems [3,4].

Soybeans (*Glycine max* L.) are nutrient-dense raw materials rich in proteins, carbohydrates, dietary fiber, vitamins, minerals, and diverse phytochemicals. Among these, polyphenols such as flavonoids, phenolic acids, and isoflavones are widely recognized for their antioxidant, antihypertensive, and cholesterol-lowering activities, which are associated with their aromatic structures and hydroxyl groups enabling radical scavenging and metal chelation [5]. Phytochemical composition varies with genotype, particularly seed coat color, with black soybeans containing higher levels of anthocyanins and isoflavones than yellow soybeans, resulting in superior antioxidant capacity. Fermentation is an effective strategy to enhance soybean functionality. Microbial enzymes degrade macromolecules and convert bound phenolics into bioavailable forms, increasing aglycone isoflavones and total phenolic content (TPC). These changes improve antioxidant and anti-inflammatory activities while reducing anti-nutritional factors [6]. Collectively, compositional characteristics and fermentation-driven transformations determine the biofunctional properties of soybean-derived products [7].

Reactive oxygen species (ROS) and reactive nitrogen species (RNS) are inevitable byproducts of cellular metabolism and can induce oxidative stress when excessively accumulated, leading to damage of proteins, lipids, and DNA. Increasing evidence links oxidative stress to hypertension, where reduced nitric oxide (NO) bioavailability, endothelial dysfunction, and activation of inflammatory and redox-sensitive pathways contribute to elevated vascular resistance [8]. Moreover, oxidative stress plays a critical role in aging and neurodegenerative diseases by promoting neuroinflammation, neuronal apoptosis, and protein aggregation [9]. Fermented foods, particularly kefir, have attracted attention due to their antioxidant and anti-inflammatory properties mediated by diverse microbial communities and metabolites, which can modulate gut microbiota and neurochemical signaling [2]. However, traditional dairy-based kefir presents limitations, including lactose intolerance, milk protein allergies, and dietary restrictions, limiting its broader application [10]. Plant-based kefir alternatives have therefore gained increasing interest. Soy milk represents a promising substrate due to its rich nutritional composition and abundance of bioactive compounds. Fermentation may enhance soybean functionality by converting macromolecules into low-molecular-weight compounds and increasing the bioavailability of peptides, phenolic acids, fatty acids, vitamins, flavonoids, minerals, and organic acids, thereby improving antioxidant and anti-inflammatory activities [11]. This study was the first to systematically investigate the effects of kefir fermentation and oxidative degradation on the physicochemical characteristics, antioxidant activity, angiotensin-converting enzyme (ACE) inhibitory capacity, and in vitro neuroprotective potential of soybean-derived polysaccharide-rich extracts from yellow and black soybeans. The study aimed to elucidate the relationships between compositional changes and biological activities through the integration of physicochemical characterization, cellular assays, and multivariate analysis. The findings highlight the dual effects of oxidative degradation, which enhanced antioxidant and ACE inhibitory activities while potentially reducing the protective effects observed in neuronal cells. These results provide new insights into the development and application of kefir-derived soybean polysaccharide-rich extracts as functional ingredients for nutraceutical and functional food applications.

## 2. Materials and Methods

### 2.1. Materials

Yellow soybeans (*Glycine max* (L.) Merr.) and black soybeans (*Glycine max* (L.) Merr. cv. “Tainan 3”) were obtained from a local supplier in Kaohsiung City, Taiwan. The kefir starter culture was provided by DK SHOP, National Kaohsiung University of Science and Technology, Kaohsiung City, Taiwan, and contained *Lactobacillus acidophilus*, *Bifidobacterium longum*, *Lactobacillus paracasei*, *Lactobacillus rhamnosus*, *Lactobacillus fermentum*, *Streptococcus thermophilus*, *Lactobacillus helveticus*, and *Kluyveromyces marxianus*. Analytical standards and reagents, including gallic acid, 2,2-diphenyl-1-picrylhydrazyl (DPPH), 2,2′-azino-bis(3-ethylbenzothiazoline-6-sulfonic acid) (ABTS), trifluoroacetic acid (TFA), ferrozine, hippuryl-histidyl-leucine, angiotensin converting enzyme (ACE), captopril, dextrans, and Bradford reagent, were purchased from Sigma-Aldrich, St. Louis, MO, USA. Cell culture reagents, including RPMI-1640 medium, fetal bovine serum, penicillin, and streptomycin, were obtained from Gibco Laboratories, Grand Island, NY, USA. All other chemicals were of analytical grade.

### 2.2. Extraction of Polysaccharides from Unfermented Soybeans

Yellow and black soybeans (30 g each) were soaked in distilled water at a solid-to-liquid ratio of 1:10 at 4 °C for 16 h. The soaked samples were homogenized with 360 mL distilled water using a homogenizer (IKA T25 digital ULTRA-TURRAX, IKA^®^-Werke GmbH and Co. KG, Staufen, Germany) at 12,000 rpm for 5 min. The homogenates were filtered three times using a sterile triple-layer filter cloth to remove insoluble residues. The filtrates were sterilized in an autoclave (TM-322, Tomin Co., Ltd., New Taipei City, Taiwan) at 121 °C for 15 min and cooled to 25 ± 2 °C. The samples were centrifuged at 12,000 rpm for 20 min at 4 °C using a refrigerated centrifuge (5810 R, Eppendorf, Hamburg, Germany). The supernatants were freeze-dried using a lyophilizer (FDU-2100, Tokyo Rikakikai Co., Ltd., Tokyo, Japan) to obtain unfermented soybean polysaccharide-rich extracts, designated as S and B, respectively.

### 2.3. Extraction of Polysaccharides from Fermented Soybeans

Fermented samples were prepared with modifications based on Tiss et al. (2020) [12]. Yellow and black soybeans (30 g each) were processed under the same soaking, homogenization, filtration, and sterilization conditions described above. After cooling, kefir starter culture at 3% was inoculated into the soybean milks and mixed thoroughly. Fermentation was conducted at 35 °C for 48 h. The fermented samples were centrifuged at 12,000 rpm for 20 min at 25 °C, followed by a second centrifugation at 12,000 rpm for 30 min at 4 °C to remove residual solids. The supernatants were collected, frozen at −80 °C, and freeze-dried to obtain fermented soybean polysaccharide-rich extracts, designated as S-F and B-F, respectively.

### 2.4. Preparation of Degraded Fermented Soybean Polysaccharides

Degraded fermented samples were prepared with modifications based on Huang et al. (2018) [13]. Each lyophilized fermented sample (0.4 g) was dissolved in 40 mL of distilled water, followed by the addition of 10 mM ascorbic acid and 10 mM hydrogen peroxide. The mixtures were incubated with shaking at 25 °C for 16 h under ambient conditions to facilitate degradation. Subsequently, the degraded samples were centrifuged at 12,000 rpm for 30 min at 4 °C to remove residual solids. The resulting supernatants were then frozen at −80 °C and lyophilized to obtain degraded fermented polysaccharide-rich extracts, designated as S-FD and B-FD, respectively.

### 2.5. Determination of Chemical Composition and Bioactive Compounds

Total sugar content was determined using the phenol-sulfuric acid method with D-(+)-galactose as a standard [14]. Briefly, 1 mL of galactose standard solutions at different concentrations or 1 mL of soybean polysaccharide samples (0.1 mg/mL) were mixed with 0.5 mL of 5% (*w*/*v*) phenol solution. Subsequently, 2.5 mL of concentrated sulfuric acid (98%, *v*/*v*) was rapidly added within 10–20 s, followed by thorough mixing. The reaction mixture was allowed to stand at room temperature for 10 min and then incubated in a water bath at 30 °C for 20 min. After cooling to room temperature, the absorbance was measured at 490 nm using a microplate reader. The total sugar content of each sample was calculated from the galactose standard calibration curve. Reducing sugar was analyzed using the 3,5-dinitrosalicylic acid (DNS) method with dextrose as a standard [15]. Briefly, 125 μL of dextrose standard solutions at various concentrations or 125 μL of soybean polysaccharide samples (4 mg/mL) was mixed with 125 μL of DNS reagent. The mixture was thoroughly vortexed and heated in an oil bath at 100 °C for 5 min to allow color development. After cooling to room temperature, 750 μL of deionized water was added to each reaction mixture and mixed thoroughly. The absorbance was measured at 540 nm using a microplate reader. The reducing sugar concentration was calculated from the dextrose standard calibration curve. Protein content was quantified using the Bradford assay with bovine serum albumin (BSA) as a standard [16]. Briefly, 20 μL of BSA standard solutions at various concentrations or 20 μL of soybean polysaccharide samples (1 mg/mL) was mixed with 1 mL of Bradford reagent and thoroughly vortexed. The reaction mixtures were incubated at room temperature in the dark for 15 min to allow color development. The absorbance was measured at 595 nm using a microplate reader. Protein concentrations were calculated from the BSA standard calibration curve. TPC was determined using the Folin–Ciocalteu method with gallic acid as a standard [14]. Briefly, 125 μL of gallic acid standard solutions at various concentrations or 125 μL of soybean polysaccharide samples (2 mg/mL) was mixed with Folin–Ciocalteu reagent. After thorough mixing, the reaction mixtures were incubated at room temperature in the dark for 5 min. Subsequently, 500 μL of 7.5% (*w*/*v*) sodium carbonate (Na_2_CO_3_) solution was added, and the mixtures were further incubated in the dark at room temperature for 2 h to allow color development. The absorbance was measured at 760 nm using a microplate reader. The TPC was calculated from the gallic acid standard calibration curve. Total flavonoid content (TFC) was evaluated using the 2-aminoethoxydiphenyl borate (2-APB) method with quercetin as a standard [17]. Briefly, quercetin standard solutions at various concentrations or soybean polysaccharide samples (2 mg/mL) were dissolved in methanol and thoroughly mixed. The mixtures were centrifuged at 3000 rpm for 10 min, and 1 mL of the resulting supernatant was collected. Subsequently, 50 μL of 1% (*w*/*v*) 2-APB reagent was added and mixed thoroughly to allow complex formation between flavonoids and the reagent. The absorbance was measured at 404 nm using a microplate reader. The flavonoid concentration was calculated from the quercetin standard calibration curve. All measurements were performed in triplicate, and the results were expressed as a percentage of dry weight (% DW).

### 2.6. Monosaccharide Composition Determination

Monosaccharide composition was analyzed using a high-performance liquid chromatography (HPLC) system equipped with an Inspire™ C18 column (250 × 4.6 mm, 5 μm, DIKMA^®^, Beijing, China). The mobile phase consisted of triethylamine in ammonium acetate buffer and acetonitrile. Gradient elution was performed from 10% to 45% Buffer B over 69 min, followed by 100% Buffer B at 70 min and re-equilibration to 45% until 80 min. The flow rate was 1 mL/min, and detection was carried out at 245 nm. Samples of soybean polysaccharide (0.01 g) were hydrolyzed with 2 M TFA at 110 °C for 4 h, neutralized, and diluted. The hydrolysates were derivatized with 1-phenyl-3-methyl-5-pyrazolone under alkaline conditions at 70 °C for 100 min. After reaction, hydrochloric acid was added, followed by chloroform extraction. The aqueous phase was collected, filtered, and analyzed. Monosaccharides were identified and quantified using standards including rhamnose, glucose, glucuronic acid, galactose, xylose, mannose, and galacturonic acid. Results were expressed as molar ratios.

### 2.7. Determination of Phenolic Composition

Phenolic composition was determined according to a previously reported method [18]. Briefly, phenolic compounds were analyzed using an HPLC system equipped with an Inspire™ C18 column (250 × 4.6 mm, 5 μm; DIKMA^®^, China). The mobile phase consisted of 2% (*v*/*v*) acetic acid in water (eluent A) and 0.5% (*v*/*v*) acetic acid in a water/acetonitrile mixture (50:50, *v*/*v*; eluent B). Gradient elution was performed as follows: 0–50 min, 20–55% eluent B; 50–60 min, 55–100% eluent B; and 60–65 min, 100–20% eluent B. The flow rate was maintained at 1.0 mL/min, the injection volume was 20 μL, and detection was carried out at 280 nm. The column temperature was maintained at 25 °C. For sample preparation, extracts were dissolved in methanol and 6 N HCl (4:1, *v*/*v*) to obtain a final concentration of 5 mg/mL. The mixtures were incubated at 90 °C for 2 h and subsequently filtered through a 0.45 μm PTFE membrane filter prior to HPLC analysis. Phenolic compounds were identified using authentic standards, including chlorogenic acid, gallic acid, coumaric acid, tannic acid, homogentisic acid, vanillic acid, catechin, epicatechin, catechin gallate (CG), epicatechin gallate (ECG), epigallocatechin gallate (EGCG), rutin, ellagic acid, ferulic acid, daidzein, mangiferin, quercetin, and genistein (Sigma-Aldrich, St. Louis, MO, USA). Compound identification was performed by comparing the retention times of sample peaks with those of the corresponding authentic standards analyzed under identical chromatographic conditions.

### 2.8. Fourier Transform Infrared (FTIR) Spectroscopy

Soybean polysaccharide samples were ground with potassium bromide at a ratio of 1:50 and dried at 50 °C. The mixtures were compressed into pellets and analyzed using an FTIR spectrometer. Spectra were recorded over a range of 4000 to 400 cm^−1^ with 64 scans and a resolution of 16 cm^−1^, using potassium bromide as the background.

### 2.9. Molecular Weight Analysis

The molecular weight distribution of the polysaccharide samples was analyzed by size exclusion chromatography (SEC) using a Superdex 200 gel filtration column (10 × 300 mm; GE Healthcare, Chicago, IL, USA) coupled to a SHIMADZU HPLC system (Shimadzu, Kyoto, Japan) equipped with a refractive index detector. Dextran standards (50, 150, and 670 kDa) were used for calibration. Polysaccharide samples were dissolved at a concentration of 2 mg/mL, filtered through a membrane filter, and injected at a volume of 20 μL. Elution was carried out using 0.2 M sodium chloride as the mobile phase at a flow rate of 0.3 mL per min. Molecular weight distribution was estimated based on the retention times of the dextran standards.

### 2.10. ACE Inhibitory Activity

ACE inhibitory activity was determined by quantifying hippuric acid released from hippuryl histidyl leucine using a modified method [16]. HPLC analysis was performed using an Inspire™ C18 column (250 × 4.6 mm, 5 μm; DIKMA^®^, Beijing, China). The mobile phase consisted of 50% methanol containing 0.1% TFA, delivered at a flow rate of 0.8 mL/min, and a column temperature of 25 °C. The eluted HA was monitored at 228 nm. For the ACE reaction, 40 μL of ACE solution (0.1 U/mL) was mixed with 40 μL of 0.1 M borate buffer containing 0.3 M NaCl (pH 8.3). Subsequently, 40 μL of 5.6 mM HHL solution was added, and the reaction mixture was thoroughly mixed and incubated in a water bath at 37 °C for 50 min. The enzymatic reaction was terminated by the addition of 135 μL of 1 N HCl. This mixture served as the negative control. For the positive control, 40 μL of captopril solution (5 μM) was used in place of the borate buffer. For the experimental groups, 40 μL of soybean polysaccharide samples (2 mg/mL) was substituted for the borate buffer. The amount of HA produced was quantified by HPLC, and the ACE inhibitory activity was calculated based on the reduction in HA formation relative to the control. All measurements were performed in triplicate.

### 2.11. DPPH Radical Scavenging Activity

The DPPH radical scavenging activity was evaluated according to a previously reported method [19], using butylated hydroxyanisole (BHA) as the reference standard. Briefly, 50 μL of BHA solution at various concentrations or soybean polysaccharide samples at various concentrations were mixed with 150 μL of a 0.1 mM DPPH methanolic solution. The reaction mixtures were incubated in the absence of light at ambient temperature for 30 min to ensure sufficient interaction between the radicals and antioxidants. Following incubation, the absorbance was recorded at 517 nm using a microplate reader. The percentage of radical scavenging activity was calculated according to the following equation:

Scavenging rate (%) = [1 − (A_sample_/A_blank_)] × 100.

Results were expressed as IC_50_ values, representing the concentration required to scavenge 50% of DPPH radicals.

### 2.12. ABTS Radical Scavenging Activity

The ABTS radical scavenging capacity was determined according to a previously reported method [19], using BHA as a positive control. The ABTS radical cation was first generated by reacting ABTS stock solution with potassium persulfate, followed by incubation in the dark at room temperature for 12 to 16 h to allow complete radical formation. Prior to analysis, the resulting solution was diluted with methanol to obtain an absorbance of approximately 0.70 at 734 nm. For the assay, 100 μL of the diluted ABTS solution was combined with 100 μL of BHA solution at various concentrations or soybean polysaccharide samples at various concentrations. The reaction mixtures were kept in the dark for 6 min at room temperature. The decrease in absorbance was then measured at 734 nm using a microplate reader. The percentage of ABTS radical scavenging activity was calculated according to the following equation:

Scavenging rate (%) = [1 − (A_sample_/A_blank_)] × 100.

Results were expressed as IC_50_ values.

### 2.13. Ferrous Iron Chelating Activity

Ferrous ion chelating activity was evaluated according to a previously reported method [19], using ethylenediaminetetraacetic acid (EDTA) as a reference standard. In brief, 200 μL of EDTA solution at various concentrations or soybean polysaccharide samples at various concentrations were combined with 740 μL of methanol and 20 μL of ferrous chloride solution. After thorough mixing, 40 μL of ferrozine reagent was added to initiate the formation of the ferrozine–Fe^2+^ complex. The reaction mixture was then allowed to stand at room temperature for 10 min to ensure complete complexation. The absorbance was subsequently measured at 562 nm using a spectrophotometer. The metal chelating activity was calculated according to the following equation:

Chelating rate (%) = [1 − (A_sample_/A_blank_)] × 100.

Results were expressed as IC_50_ values.

### 2.14. Ferric Reducing Antioxidant Power

Ferric reducing antioxidant capacity was evaluated according to a previously reported method [19], using BHA as a reference standard. Briefly, 100 μL of BHA solution (12 mg/mL) or soybean polysaccharide samples (12 mg/mL) were combined with phosphate buffer and potassium ferricyanide, followed by incubation at 50 °C for 20 min to facilitate the reduction reaction. After cooling to room temperature, trichloroacetic acid was added to terminate the reaction, and the mixture was centrifuged to obtain a clear supernatant. An aliquot of the supernatant was then mixed with distilled water and ferric chloride, and the resulting solution was allowed to react for 10 min. The absorbance was subsequently recorded at 700 nm using a spectrophotometer, and increased absorbance was indicative of greater reducing power.

### 2.15. Cell Culture and Viability Assays

The human neuroblastoma cell line SH-SY5Y (ATCC^®^ CRL-2266™, Manassas, VA, USA) was obtained from the Food Industry Research and Development Institute (Hsinchu, Taiwan). The cell culture and viability assays were performed according to a previously reported protocol [20]. Briefly, cells were cultured in a 1:1 mixture of Dulbecco’s Modified Eagle’s Medium and Ham’s F-12 supplemented with 10% fetal bovine serum, penicillin, and streptomycin at 37 °C in 5% CO_2_, with medium refreshed every 2 to 3 days. For the viability assay, cells were seeded in 96-well plates at 1.5 × 10^4^ cells per well and incubated for 8 h. After treatment with samples for 24 h, MTT reagent was added and incubated for 4 h. Formazan crystals were dissolved in isopropanol, and absorbance was measured at 490 nm. Cell viability was expressed relative to the control.

### 2.16. Annexin V and Propidium Iodide (PI) Assay

The annexin V and PI assay was performed according to a previously reported protocol [14]. In brief, SH-SY5Y cells were seeded in 6 cm dishes at 4 × 10^5^ cells per well and incubated at 37 °C in 5% CO_2_ for 24 h. Cells were treated with soybean polysaccharide samples for 24 h, followed by exposure to 50 μM rotenone (ROT) for an additional 24 h. After treatment, cells were collected, washed with phosphate-buffered saline, and resuspended in binding buffer. Annexin V and PI staining was performed according to the manufacturer’s instructions, and samples were incubated in the dark for 15 min. Apoptosis was analyzed using a BD Accuri C6 flow cytometer (BD Biosciences, Becton, Dickinson and Company, Franklin Lakes, NJ, USA), with 10,000 events recorded per sample and data processed using the instrument software.

### 2.17. Statistical Analysis

Statistical analyses were performed using SPSS software (version 12.0). Differences among groups were evaluated by one-way analysis of variance, followed by Duncan’s multiple range test for post hoc comparisons. All data are presented as the mean ± standard deviation based on at least three independent experiments. Statistical significance was defined at a probability level of *p* < 0.05.

## 3. Results and Discussion

### 3.1. Molecular Weight, Chemical Composition, and Monosaccharide Composition Analyses of S-F, S-FD, B-F, and B-FD

The molecular weight distributions of the fermented polysaccharide-rich extracts (S-F and B-F) and their degraded derivatives (S-FD and B-FD) were determined by SEC using a Superdex 200 gel filtration column, and the results are summarized in Table 1. In the present study, the average molecular weights of S-F, S-FD, B-F, and B-FD were 263.6, 88.6, 232.7, and 95.2 kDa, respectively. While S-F and B-F exhibited similar molecular weights, S-FD and B-FD showed markedly lower values, consistent with the reduction in molecular size following oxidative degradation. Previous studies have suggested that polysaccharides with higher molecular weights tend to exhibit lower solubility, which may hinder their cellular uptake and subsequently limit their biological activities [21]. In addition, Wang et al. (2010) [22] reported that antioxidant activity is closely associated with molecular weight, with lower-molecular-weight polysaccharides generally demonstrating enhanced antioxidant potential. Given the distinct molecular weight distributions among S-F, S-FD, B-F, and B-FD, their corresponding biological activities warrant further investigation.

The chemical compositions of S-F, S-FD, B-F, and B-FD are presented in Table 1. The total sugar contents of S-F, S-FD, B-F, and B-FD were 29.3 ± 0.3%, 10.5 ± 1.9%, 22.5 ± 0.4%, and 13.8 ± 0.6%, respectively, which were markedly lower than those of unfermented yellow soybean polysaccharide (S, 68.4 ± 2.8%) and unfermented black soybean polysaccharide (B, 73.8 ± 1.4%). The reduction in total sugar content after fermentation and oxidative degradation may be attributed to enzymatic depolymerization and microbial utilization of released sugars as carbon and energy sources [23]. Oxidative degradation further cleaves glycosidic linkages, generating low-molecular-weight compounds that may not be fully detected by conventional assays [24]. In contrast, reducing sugar contents were significantly higher in S-FD and B-FD than in their corresponding fermented samples, likely due to glycosidic bond cleavage and the release of free aldehyde and ketone groups during degradation [25]. Protein content showed no substantial changes after degradation. The degradation process significantly increased total phenolic and total flavonoid contents in S-FD and B-FD (*p* < 0.05). Since soybean isoflavones are predominantly present in glycosylated forms [26]. Oxidative treatment may disrupt cell wall structures and cleave glycosidic linkages, thereby facilitating the release of bound phenolic compounds and flavonoids [25]. These findings suggest that fermentation and oxidative degradation synergistically alter the chemical composition and bioactive compound profiles of soybean-derived kefir polysaccharide-rich extracts.

During fermentation, kefir-associated microorganisms produce carbohydrate-active enzymes, particularly β-glucosidase, which hydrolyze soybean polysaccharides and alter monosaccharide composition [27]. Variations in monosaccharide profiles may also result from differences in microbial consortia, raw materials, and processing conditions [28,29]. The monosaccharide compositions of S-F and S-FD are presented in Table 1. Mannose and glucuronic acid were the predominant monosaccharides in both samples. Following oxidative degradation, the relative contents of rhamnose, galacturonic acid, galactose, and xylose increased in S-FD compared with S-F. Similar changes in monosaccharide profiles after oxidative degradation have been reported previously and are generally associated with alterations in polysaccharide composition during depolymerization [30,31]. These results indicate that oxidative degradation significantly modified the monosaccharide composition of yellow soybean-derived polysaccharide-rich extracts. For the black soybean group, mannose and rhamnose were the predominant monosaccharides in both B-F and B-FD (Table 1), differing from the yellow soybean group. The relatively high rhamnose content may be related to the compositional characteristics of black soybean polysaccharides, which have been reported to contain pectic polysaccharides enriched in rhamnose and galacturonic acid [32]. Following oxidative degradation, B-FD exhibited higher proportions of rhamnose, glucuronic acid, galacturonic acid, glucose, and galactose than B-F. Depolymerization-induced alterations in monosaccharide profiles have been widely reported and are generally attributed to changes in polysaccharide composition and the relative abundance of constituent monosaccharides [33,34,35]. The increased proportions of uronic acids and neutral sugars observed in B-FD indicate that oxidative degradation significantly modified the monosaccharide composition of the polysaccharide-rich extract. Overall, these results demonstrate that oxidative degradation altered the monosaccharide distribution of soybean-derived polysaccharides and increased the relative abundance of several monosaccharide components.

### 3.2. Phenolic Component Analysis of S-F, S-FD, B-F, and B-FD

In leguminous plants, many phenolic compounds exist in insoluble forms bound to cell wall components. During fermentation, carbohydrate-degrading enzymes facilitate the release of these bound phenolics into soluble forms [36]. The phenolic compositions of S-F, S-FD, B-F, and B-FD are summarized in Table 2. In the present study, EGCG, vanillic acid, and tannic acid were the predominant phenolic compounds detected in all groups. Previous studies have reported syringic acid, ferulic acid, and vanillic acid as major phenolics in soybean seeds [37]. Oxidative degradation may contribute to changes in the phenolic profiles of polysaccharide-rich extracts derived from both yellow and black soybeans. In the yellow soybean group, degradation increased the contents of vanillic acid and homogentisic acid, whereas in the black soybean group, increased levels of EGCG and vanillic acid were observed. These changes may be associated with the disruption of the polysaccharide matrix during oxidative degradation, which could facilitate the release of phenolic compounds previously associated with or entrapped within it. In addition, polysaccharide depolymerization may improve extractability and increase the detectable levels of certain phenolic compounds. A limitation of the present study is that phenolic compounds were identified and quantified by HPLC using authentic standards, whereas LC-MS/MS confirmation was not performed. Therefore, the identification of certain compounds should be interpreted with caution. For example, the relatively high levels of EGCG detected in S-F and B-FD warrant careful consideration, as EGCG is generally recognized as a major catechin in tea rather than a characteristic phenolic constituent of soybeans. EGCG belongs to the catechin family of flavan-3-ols and shares similar chromatographic characteristics with several structurally related phenolic compounds [38]. Therefore, identification based solely on HPLC retention time may not completely exclude the possibility of co-eluting compounds. Future studies employing LC-MS/MS would provide greater confidence in compound identification and facilitate the detection of minor fermentation-derived metabolites. Overall, the present study demonstrated differences in the phenolic profiles of S-F, S-FD, B-F, and B-FD as determined by HPLC analysis. Nevertheless, the factors contributing to these variations were not examined in the present study. Furthermore, because compound identification was not confirmed by LC-MS/MS, the assignment of certain phenolic compounds should be regarded as tentative. Future studies using advanced analytical approaches are needed to verify compound identities, improve the characterization of phenolic constituents, and facilitate a more comprehensive evaluation of their potential relevance to the observed properties of soybean-derived kefir extracts.

### 3.3. FTIR Analysis for S-F, S-FD, B-F, and B-FD

**Figure 1:**
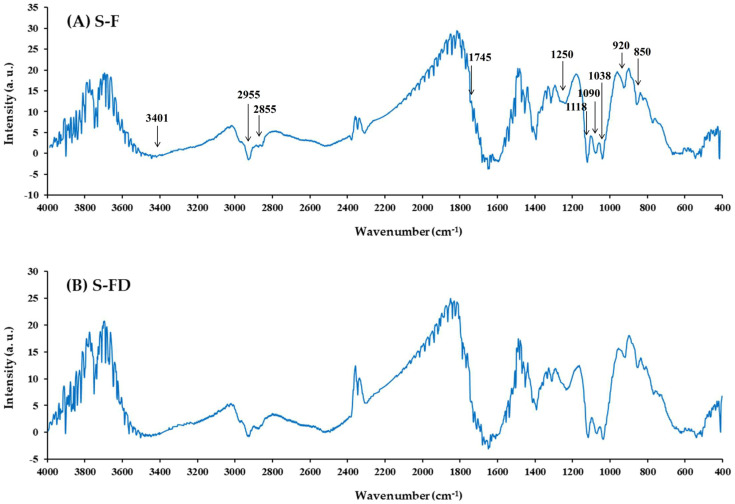

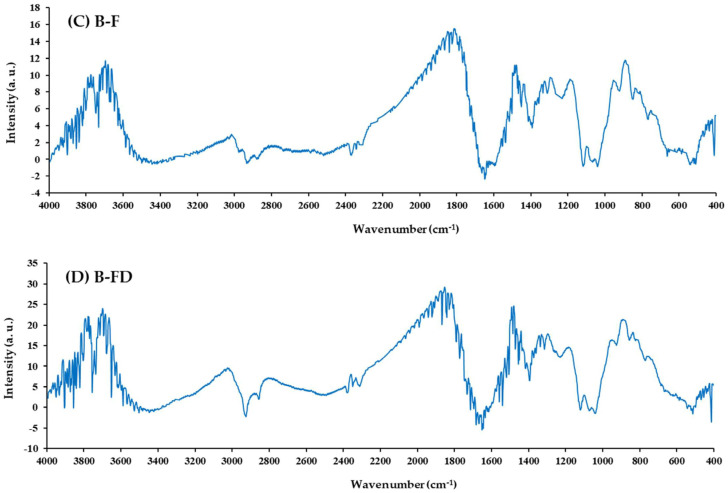
Fourier transform infrared (FTIR) spectra of kefir-fermented soybean polysaccharide-rich extracts (S-F (**A**) and B-F (**C**)) and their oxidatively degraded products (S-FD (**B**) and B-FD (**D**)). All samples exhibited characteristic polysaccharide absorption bands at approximately 3401 cm^−1^ (O–H stretching), 2955–2855 cm^−1^ (C–H stretching), 1745 cm^−1^ (carbonyl-related vibration), and 1250–1038 cm^−1^ (glycosidic linkage and carbohydrate fingerprint region). The characteristic peaks at 1118 and 1038 cm^−1^ remained evident after oxidative degradation, indicating preservation of the core polysaccharide backbone structure.

FTIR analysis was performed to evaluate structural changes in S-F, S-FD, B-F, and B-FD after oxidative degradation (Figure 1). All samples exhibited a broad absorption band around 3401 cm^−1^, corresponding to O–H stretching vibrations associated with hydrogen bonding in polysaccharides [39]. The absorption band near 2955 cm^−1^ was attributed to the stretching vibrations of aliphatic C–H bonds in the polysaccharide chains, which are characteristic of carbohydrate structures [29,40]. The band at approximately 2855 cm^−1^ was attributed to the symmetric stretching vibration of aliphatic CH_2_ groups. The absorption peak near 1745 cm^−1^ was assigned to the stretching vibration of carbonyl (C=O) groups, which may be associated with esterified or acetylated components in the polysaccharide-rich extracts [41]. Compared with fermented samples (S-F and B-F), the degraded samples (S-FD and B-FD) showed slight variations in the intensities of peaks around 1745 cm^−1^ and 1250 cm^−1^. The band near 1745 cm^−1^ is associated with carbonyl groups, suggesting that oxidative degradation may promote partial oxidation of hydroxyl groups and formation of carboxyl-containing structures [41]. In addition, the enhanced absorption around 1250 cm^−1^ may be related to C–O–C or C–O–P stretching vibrations, indicating structural modifications induced during oxidation [42]. The carbohydrate fingerprint region (1200–1000 cm^−1^), including characteristic peaks at 1118, 1090, and 1038 cm^−1^, remained evident in all samples [43,44,45]. These bands are attributed to glycosidic linkages and pyranose ring vibrations, indicating that the core polysaccharide backbone structure was retained after degradation. Furthermore, the absorption peaks around 920 cm^−1^ and 850 cm^−1^, associated with glycosidic linkage patterns and α-anomeric configurations, were preserved [43,46]. Overall, FTIR results suggest that oxidative degradation mainly altered specific functional group intensities without disrupting the fundamental polysaccharide framework or generating distinct new functional groups.

### 3.4. Antihypertensive Potential for S, B, S-F, S-FD, B-F, and B-FD

**Figure 2:**
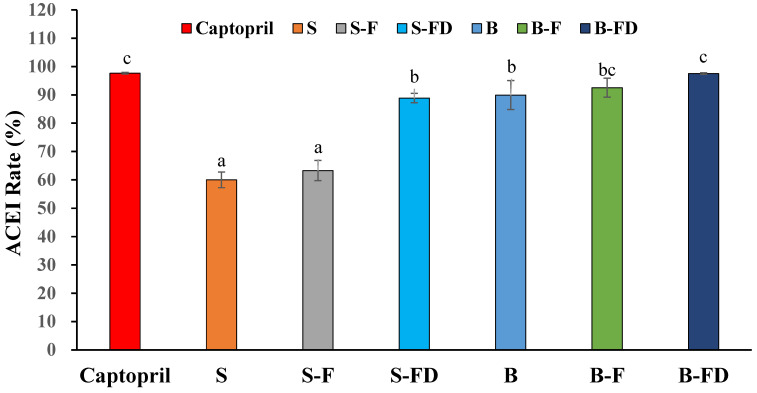
Angiotensin converting enzyme (ACE) inhibitory activities of S, S-F, S-FD, B, B-F, and B-FD at a concentration of 2 mg/mL. Kefir fermentation increased ACE inhibitory activity in both yellow and black soybean polysaccharide-rich extracts, and oxidative degradation further enhanced the inhibitory effect. Captopril (5 μM) was used as the positive control. Values are presented as mean ± SD (*n* = 3). Different superscript letters indicate significant differences among samples (*p* < 0.05).

ACE catalyzes the conversion of angiotensin I into angiotensin II and simultaneously degrades bradykinin, a potent vasodilator, thereby contributing to increased vascular tone and elevated blood pressure [47]. Therefore, inhibition of ACE activity is considered an effective strategy for alleviating hypertension. As illustrated in Figure 2, all soybean-derived extracts exhibited measurable ACE inhibitory activity, although substantial differences were observed among treatments. The ACE inhibition rates of unfermented yellow soybean extract (S) and fermented yellow soybean extract (S-F) were approximately 60.0 ± 2.7% and 63.3 ± 3.5%, respectively, with no significant difference between the two groups (*p* = 0.359). In contrast, oxidative degradation markedly enhanced ACE inhibitory activity in the yellow soybean group, as evidenced by the significantly higher inhibition rate of S-FD (88.9 ± 1.6%; *p* = 0.00075 as compared to S-F). A similar trend was observed in the black soybean group. Unfermented black soybean extract (B) exhibited an ACE inhibition rate of 89.9 ± 5.1%, whereas fermentation slightly increased the activity to 92.5 ± 3.3% in B-F (*p* = 0.577). The highest ACE inhibitory activity was observed in B-FD (97.5 ± 0.3%), which was statistically comparable to the positive control captopril (*p* = 0.694). The enhanced ACE inhibitory activity observed after oxidative degradation may be associated with compositional changes occurring during the degradation process. In the present study, oxidative degradation significantly reduced molecular weight and increased reducing sugar, total phenolic, and flavonoid contents (Table 1). Previous studies have reported that reducing the molecular weight of polysaccharides may enhance their biological activities by improving solubility and increasing the accessibility of functional groups involved in interactions with biological targets [48]. Furthermore, oxidative degradation can facilitate the release of phenolic compounds that are originally entrapped within the polysaccharide matrix, thereby increasing their extractability and detectable concentrations [25]. Polyphenolic compounds are well recognized for their antioxidant properties and have also been reported to exhibit ACE inhibitory activity. Previous studies suggest that phenolic acids and flavonoids may contribute to ACE inhibition through interactions with the enzyme and its catalytic region through hydrogen bonding, hydrophobic interactions, and metal-ion coordination, ultimately reducing enzyme activity [49]. Therefore, the elevated phenolic and flavonoid contents observed in S-FD and B-FD (Table 1) may partially contribute to the enhanced ACE inhibition observed in these samples. The superior ACE inhibitory activity observed in the black soybean-derived extracts may be associated, at least in part, with differences in chemical composition. Black soybeans are known to contain higher levels of phenolic compounds, flavonoids, anthocyanins, and other antioxidant constituents than yellow soybeans [7,50], which may contribute to their enhanced biological activities. Consistent with this observation, B-F and B-FD exhibited higher ACE inhibition rates than their corresponding yellow soybean counterparts.

Several limitations of the present study should be acknowledged. The specific compounds responsible for the observed ACE inhibitory activity were not identified. Although compositional analyses revealed significant changes in polysaccharides and phenolic constituents following fermentation and oxidative degradation, the individual contributors to ACE inhibition remain unclear. Comprehensive identification of active compounds is challenging because soybean-derived kefir extracts consist of complex mixtures of polysaccharides, phenolic compounds, isoflavonoids, organic acids, and other fermentation-derived metabolites. The isolation and characterization of individual ACE-inhibitory constituents would require extensive chromatographic purification, together with advanced spectroscopic and mass spectrometric analyses. Therefore, further studies are warranted to identify the principal compounds responsible for the observed ACE inhibitory activity and to elucidate the mechanisms underlying their effects.

### 3.5. Antioxidant Activities for S-F, S-FD, B-F, and B-FD

The antioxidant activities of S-F, S-FD, B-F, and B-FD were evaluated using DPPH, ABTS^•+^, reducing power, and ferrous-ion chelating assays (Table 3). In the DPPH assay, S-FD and B-FD exhibited markedly lower IC_50_ values (1.12 ± 0.03 and 1.07 ± 0.02 mg/mL, respectively) than S-F (5.60 ± 0.10 mg/mL) and B-F (3.43 ± 0.09 mg/mL), indicating enhanced radical-scavenging activity after oxidative degradation. This improvement is likely associated with the reduced molecular weight (Table 1), which may increase solubility and facilitate electron or hydrogen donation. Similar findings have been reported for low-molecular-weight polysaccharides, which exhibit improved antioxidant activity due to greater accessibility of active functional groups [51]. However, all samples remained less active than BHA. In the ABTS^•+^ scavenging assay, B-F (IC_50_ = 0.237 ± 0.003 mg/mL) exhibited stronger activity than S-F (IC_50_ = 0.631 ± 0.023 mg/mL), while oxidative degradation significantly enhanced the activity of both samples, with S-FD and B-FD showing IC_50_ values of approximately 0.075 mg/mL. Because the ABTS assay primarily involves electron-transfer mechanisms and is applicable in both aqueous and organic systems [52], the enhanced activity may be attributed to improved accessibility of electron-donating functional groups following degradation. However, all samples remained less active than BHA.

In the ferrous-ion chelating assay, S-FD and B-FD exhibited significantly stronger activity, with IC_50_ values of 1.32 ± 0.01 and 1.24 ± 0.02 mg/mL, respectively, compared with S-F (7.75 ± 0.12 mg/mL) and B-F (3.83 ± 0.01 mg/mL). Although EDTA exhibited the strongest ferrous-ion chelating activity among all tested samples, oxidative degradation significantly improved the metal-chelating capacity of both yellow and black soybean-derived polysaccharide-rich extracts compared with their corresponding undegraded counterparts. Ferrous-ion chelation is an important antioxidant mechanism because Fe^2+^ can catalyze the generation of highly reactive hydroxyl radicals through Fenton reactions. Chelation of Fe^2+^ may therefore reduce radical formation and subsequent oxidative damage [53]. In the present study, oxidative degradation enhanced the ferrous-ion chelating activity of both soybean-derived polysaccharide-rich extracts. This enhancement was observed alongside a reduction in molecular weight and an increase in reducing sugar content following oxidative degradation (Table 1). Previous studies have suggested that depolymerization may alter the physicochemical properties of polysaccharides and influence their metal-binding capacity [54]. Nevertheless, the relationship between these compositional changes and the observed chelating activity was not directly examined in the present study. The reducing power assay further supported these findings. At 12 mg/mL, S-FD and B-FD exhibited significantly higher absorbance values (1.40 ± 0.06 and 1.68 ± 0.02, respectively) than S-F (0.37 ± 0.00) and B-F (0.34 ± 0.01), indicating enhanced electron-donating capacity. Reducing power reflects the ability of antioxidants to donate electrons and terminate radical chain reactions. The increased reducing power observed after degradation is consistent with previous reports demonstrating that lower-molecular-weight polysaccharides possess improved redox properties [52,55]. Among all samples, B-FD exhibited the highest reducing power, indicating that oxidative degradation was particularly effective in enhancing the electron-donating capacity of black soybean-derived polysaccharide-rich extracts. Overall, oxidative degradation significantly enhanced the antioxidant activities of fermented yellow soybean and black soybean polysaccharide-rich extracts, as reflected by improved radical-scavenging activity, reducing power, and ferrous-ion chelating capacity. These observations coincided with changes in molecular weight and compositional characteristics following oxidative degradation. Further studies are required to clarify the factors contributing to the enhanced antioxidant activity. Nevertheless, the results indicate that soybean-derived kefir polysaccharide-rich extracts may have potential for use as functional ingredients in food and nutraceutical applications associated with antioxidant properties.

### 3.6. Neuronal Protection Potential in SH-SY5Y Cells for S-F, S-FD, B-F, and B-FD

**Figure 3:**
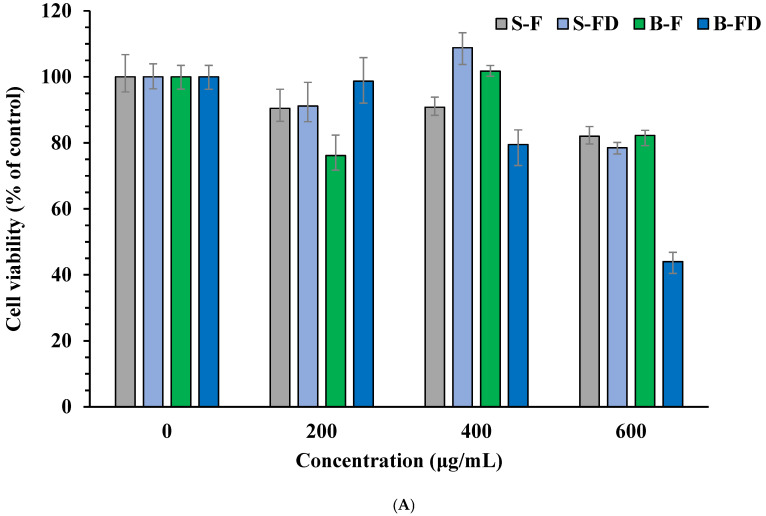

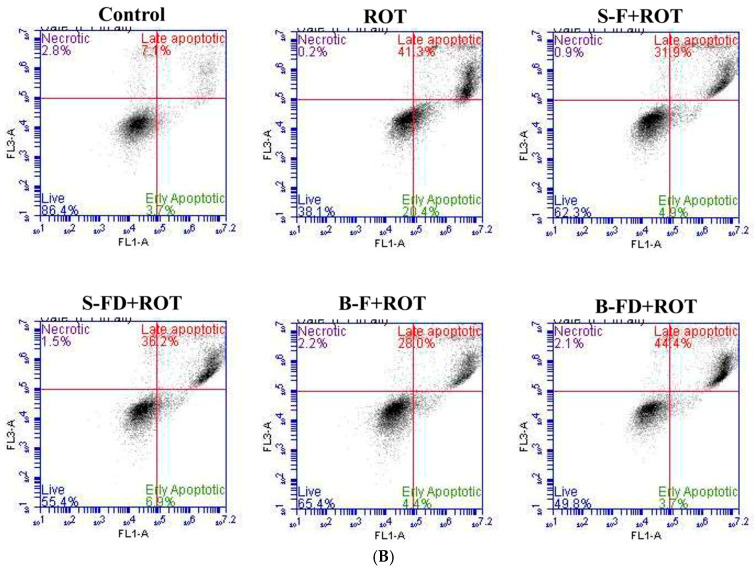

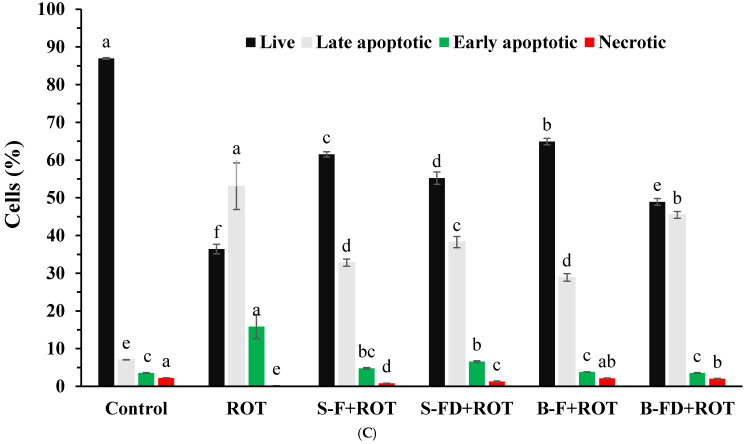
Effects of soybean-derived kefir polysaccharide-rich extracts on cell viability and ROT-induced apoptosis in SH-SY5Y human neuroblastoma cells. (**A**) Cells were treated with S-F, S-FD, B-F, and B-FD (0–600 μg/mL) for 24 h, and cell viability was determined by the MTT assay. Cell viability remained above 80% at concentrations ranging from 0 to 400 μg/mL. (**B**) Cells were pretreated with S-F, S-FD, B-F, or B-FD (200 μg/mL) for 24 h and subsequently exposed to 50 μM rotenone (ROT) for an additional 24 h. Apoptotic cell populations were analyzed by flow cytometry using Annexin V-FITC/propidium iodide (PI) double staining. (**C**) Quantitative analysis of viable, early apoptotic, late apoptotic, and necrotic cell populations. Pretreatment with polysaccharide-rich extracts increased the proportion of viable cells and reduced ROT-induced apoptotic cells, indicating protective potentials against oxidative stress-related neuronal injury. Data were analyzed using BD Accuri C6 software (version 1.0.264.21; BD Biosciences, Becton, Dickinson and Company, Franklin Lakes, NJ, USA). and are presented as mean ± SD (*n* = 3). Columns sharing at least one identical letter are not significantly different (*p* < 0.05).

In the present study, S-F, S-FD, B-F, and B-FD were evaluated for their cytotoxic effects on SH-SY5Y cells, and the results are presented in Figure 3A. The data demonstrated that cell viability remained above 80% at concentrations ranging from 0 to 400 μg/mL, indicating that all samples exhibited low cytotoxicity within this range. This finding is consistent with recent studies reporting that moderate concentrations of natural polysaccharides generally show high biocompatibility and may even enhance cell proliferation due to their antioxidant and cytoprotective properties [56,57]. In particular, fermentation-derived polysaccharides have been shown to improve cellular redox balance and promote neuronal survival under non-stress conditions. However, as the concentration increased to 400–600 μg/mL, a noticeable decline in cell viability was observed, suggesting the onset of cytotoxic effects. A possible explanation may be associated with the accumulation of bioactive constituents, including phenolic compounds, reducing sugars, and oxidation-derived products generated during fermentation and subsequent degradation. At higher concentrations, these compounds may affect cellular physiology and contribute to the observed cytotoxicity. For example, exposure to high levels of bioactive or plant-derived compounds can shift their role from protective antioxidants to pro-oxidants, thereby exacerbating intracellular oxidative damage and impairing cell survival pathways [58]. In addition, previous studies have reported that the biological effects of polysaccharides may vary with concentration [59]. Consistent with these observations, reduced cell viability was observed at relatively high concentrations of certain soybean-derived extracts in the present study. These results suggest that the cellular responses to fermentation-derived polysaccharides and their degradation products may be influenced by concentration. Therefore, careful dose-dependent evaluation is important when assessing their biological properties and potential applications. The factors associated with the reduced cell viability observed at higher concentrations were not examined in the present study and warrant further investigation. Based on these observations, a concentration of 200 μg/mL was selected for subsequent neuroprotective experiments, as it represents a physiologically relevant dose that maintains high cell viability while minimizing cytotoxic risk. Similar experimental designs have been widely adopted in the recent literature to ensure that observed neuroprotective effects are not confounded by underlying cytotoxicity [60]. Collectively, these results highlight the importance of dose optimization in evaluating the biological activities of fermented polysaccharides and their derivatives and support the selection of non-toxic concentrations for further functional investigations. The effects of S-F, S-FD, B-F, and B-FD and ROT on apoptosis in SH-SY5Y cells were further evaluated using Annexin V/PI double staining, as shown in Figure 3B,C. In the control group, the majority of cells were classified as viable, with only minimal proportions of early apoptotic, late apoptotic, and necrotic cells, indicating normal cellular homeostasis. In contrast, exposure to ROT markedly reduced the percentage of live cells and significantly increased both early and late apoptotic populations, confirming the successful establishment of an oxidative stress-induced neuronal injury model. This observation is consistent with previous studies demonstrating that ROT induces mitochondrial dysfunction and excessive ROS generation, ultimately triggering apoptosis in SH-SY5Y cells [61]. Treatment with S-F notably improved cell survival compared to the ROT group, as evidenced by an increased proportion of live cells and a reduction in apoptotic populations. The decrease in both early and late apoptosis suggests that S-F exerts a protective effect against ROT-induced cytotoxicity. This protective effect may be related to the antioxidant activity of the fermented soybean-derived polysaccharide-rich extracts (Table 3). Although the underlying mechanisms were not investigated in the present study, the observed reduction in apoptotic cells may be associated with their ability to mitigate oxidative stress. Recent studies have demonstrated that enhancing mitochondrial integrity and reducing oxidative stress can effectively suppress apoptosis signaling pathways, including caspase activation and p53-mediated responses in SH-SY5Y cells [62]. Similarly, S-FD exhibited a protective effect against ROT-induced cell injury, although its effect was slightly weaker than that of S-F. The proportion of viable cells in the S-FD group was lower, whereas apoptotic cell populations were higher than those in the S-F group. This difference may be associated with compositional changes resulting from oxidative degradation; however, the specific factors responsible for this effect were not investigated in the present study. Recent studies have emphasized that changes in molecular weight and monosaccharide composition caused by oxidative degradation can significantly influence the biological activities of polysaccharides and their structure-activity relationships [34,63]. B-F also exhibited a protective effect against ROT-induced apoptosis and showed greater improvements in cell viability and reductions in apoptotic cell populations than S-F and S-FD. These differences may be related to variations in the compositional characteristics of yellow and black soybean-derived polysaccharide-rich extracts. In contrast, B-FD showed a weaker protective effect than B-F. Although cell viability remained higher than that of the ROT-treated group, the percentages of early and late apoptotic cells were higher than those observed in the S-F, S-FD, and B-F groups. These results indicate that the protective effects observed for the oxidatively degraded samples were generally lower than those of their corresponding undegraded counterparts under the experimental conditions used in this study. Overall, S-F and B-F reduced ROT-induced apoptosis in SH-SY5Y cells, with B-F exhibiting the highest proportion of viable cells and the lowest proportion of apoptotic cells among the tested samples. The differences observed among treatments may be related to variations in compositional characteristics, including molecular weight and chemical constituents; however, no direct evidence was obtained in the present study to establish such relationships. Further studies are needed to clarify the factors associated with the observed differences in cell-protective effects.

### 3.7. Principal Component Analysis (PCA) and Z-Score Heatmap Analysis

**Figure 4:**
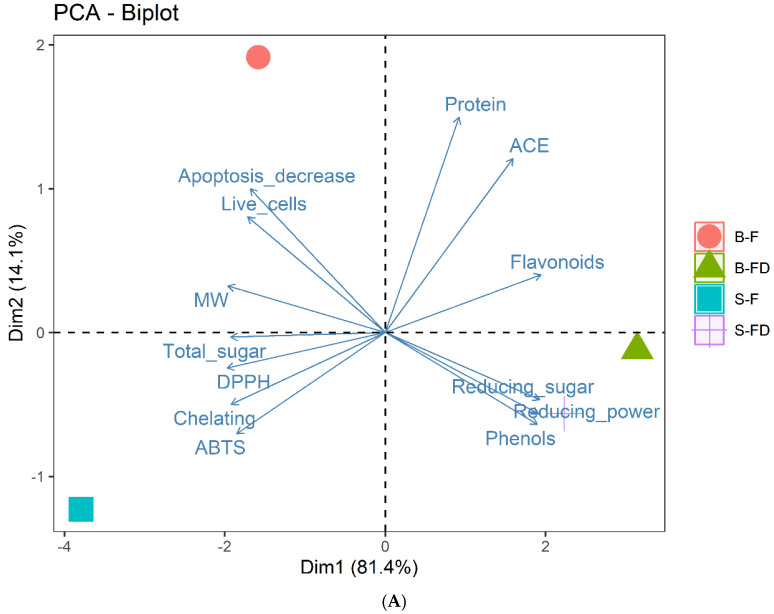

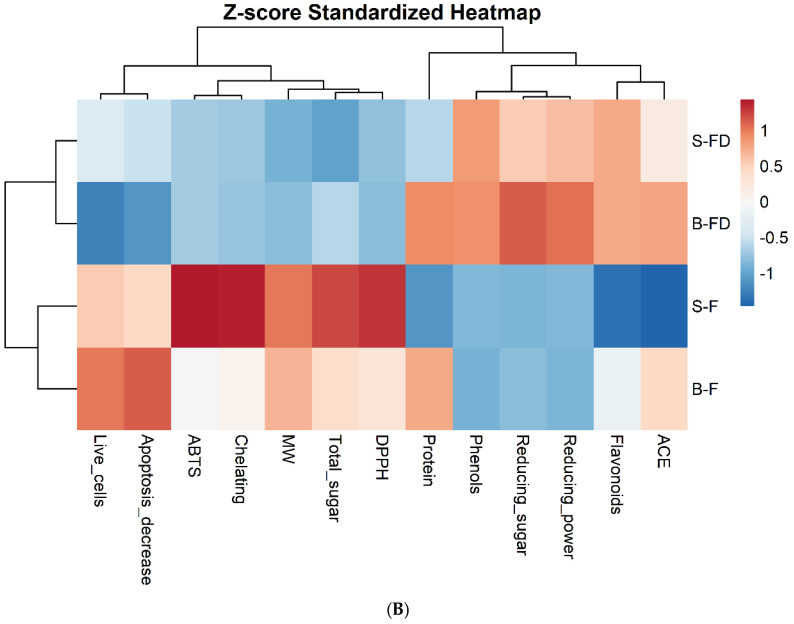
(**A**) Principal component analysis (PCA) biplot showing the relationships among physicochemical characteristics, antioxidant activities, angiotensin converting enzyme (ACE) inhibitory activity, and neuroprotective potential of soybean-derived kefir polysaccharide-rich extracts. PC1 (81.4%) primarily separated samples according to antioxidant activity, ACE inhibition, reducing sugar content, phenolic content, flavonoid content, and molecular weight, whereas PC2 (14.1%) was mainly associated with neuroprotective indicators, including increased neuronal viability and reduced rotenone (ROT)-induced apoptotic neuronal cells. Oxidatively degraded samples (S-FD and B-FD) were closely associated with enhanced antioxidant and ACE inhibitory activities, while fermented samples (S-F and B-F) were more closely related to neuroprotective potential. (**B**) Z-score standardized heatmap illustrating the clustering patterns of physicochemical properties and bioactivities. The heatmap revealed distinct grouping of degraded and non-degraded samples, indicating that oxidative degradation markedly altered compositional characteristics and bioactivity profiles. ACE, ACE inhibition; Flavonoids, total flavonoid content (%); Reducing_power, reducing power; Reducing_sugar, reducing sugar (%); Phenols, total phenolic content (%); Protein, protein (%); DPPH, DPPH (2,2-diphenyl-1-picrylhydrazyl) IC_50_; Total_sugar, total sugar (%); MW, molecular weight (kDa); Chelating, ferrous-ion chelating IC_50_; ABTS, ABTS (2,2′-azino-bis(3-ethylbenzothiazoline-6-sulfonic acid)) IC_50_; Apoptosis_decrease, decrease in apoptotic neuronal cells (%); Live_cells, increase in viable neuronal cells (%).

PCA was performed to evaluate the relationships among physicochemical properties, antioxidant activity, ACE inhibitory activity, and neuroprotective potential of soybean-derived kefir polysaccharide-rich extracts (Figure 4A). The PCA results showed clear separation among S-F, S-FD, B-F, and B-FD, indicating substantial compositional and functional differences induced by fermentation and oxidative degradation. PC1 explained 81.4% of the total variance and was positively associated with reducing power, ACE inhibition, phenolic content, flavonoid content, and reducing sugar content, while molecular weight and total sugar content showed negative loadings. These results suggest that lower-molecular-weight polysaccharide-rich extracts exhibit enhanced bioactivities. PC2 accounted for 14.1% of the variance and mainly reflected neuroprotective potential, including increased neuronal viability and reduced apoptotic neuronal cells. Fermented polysaccharide-rich extracts (S-F and B-F) were more closely associated with neuroprotective potential, whereas degraded polysaccharide-rich extracts (S-FD and B-FD) showed stronger associations with antioxidant activity and ACE inhibition. In particular, B-FD was strongly correlated with improved antioxidant and metal-chelating activities. Overall, oxidative degradation shifted polysaccharide properties from physicochemical dominance toward enhanced functional bioactivity, although this may partially reduce neuroprotective potential.

Furthermore, a Z-score standardized heatmap was constructed to systematically compare the compositional characteristics and biofunctional properties of the four polysaccharide samples (S-F, S-FD, B-F, and B-FD). Standardization enabled direct comparison across variables with different units and scales, highlighting relative enrichment or depletion patterns. The heatmap (Figure 4B) revealed two major clustering trends. First, the degraded polysaccharide-rich extracts (S-FD and B-FD) clustered together, characterized by elevated levels of reducing sugar, total phenols, total flavonoids, reducing power, and ACE inhibitory activity. These samples also exhibited lower IC_50_ values for DPPH, ABTS, and ferrous-ion chelating activity, indicating enhanced antioxidant capacity. The enrichment of these bioactive attributes is consistent with the reduced molecular weight and increased exposure of functional groups following oxidative degradation. In contrast, native polysaccharide-rich extracts (S-F and B-F) formed a separate cluster, primarily associated with higher molecular weight and total sugar content. These samples showed relatively weaker antioxidant activity but demonstrated superior neuroprotective effects, as evidenced by higher values in live neuron cell viability and reduced apoptotic cell percentages. This suggests that intact polysaccharide structures may play a more critical role in cellular protection mechanisms. At the variable level, hierarchical clustering further grouped antioxidant-related parameters (DPPH, ABTS, chelating activity, and reducing power) with phenolic and flavonoid contents, indicating strong positive correlations among these features. Conversely, molecular weight and total sugar content were inversely associated with these antioxidant indices, reinforcing the concept that oxidative degradation enhances functional bioactivity. Interestingly, neuroprotective indicators (increase in live neuron cells and decrease in apoptotic neuron cells) were more closely associated with fermented polysaccharide-rich extracts, suggesting a divergence between antioxidant potency and neuroprotective efficacy. This observation implies that while lower molecular weight polysaccharide-rich extracts exhibit superior radical scavenging and enzyme inhibitory activities, higher molecular weight fractions may retain structural motifs essential for neuronal protective potential.

Integrated PCA and Z-score standardized heatmap analyses demonstrated that oxidative degradation is a key determinant of polysaccharide bioactivity profiles. Both analyses clearly separated degraded polysaccharide-rich extracts (S-FD and B-FD) from fermented samples (S-F and B-F), mainly due to their lower molecular weight, higher reducing sugar content, and increased phenolic and flavonoid levels. These compositional changes were strongly associated with enhanced antioxidant activity, including lower DPPH, ABTS, and metal chelating IC_50_ values, greater reducing power, and improved ACE inhibitory activity. These findings are consistent with previous studies showing that low-molecular-weight polysaccharides exhibit superior antioxidant properties because of improved solubility, greater exposure of reactive groups, and enhanced electron-donating ability [52,64]. In contrast, fermented polysaccharide-rich extracts with higher molecular weight and total sugar content were more closely associated with increased neuronal viability and reduced apoptotic neuron cells, indicating stronger neuroprotective potential. Previous studies have similarly reported that high-molecular-weight polysaccharides contribute more effectively to cell-protective functions, whereas smaller fragments exhibit greater antioxidant activity [64]. In addition to changes in polysaccharide composition, kefir fermentation may generate a variety of fermentation-derived metabolites that could contribute to the observed biological activities. Previous studies have reported that kefir-associated microorganisms can produce a wide range of metabolites during fermentation, including organic acids, exopolysaccharides, vitamins, and transformed phenolic compounds, depending on the fermentation substrate and microbial community composition [65]. These metabolites have been associated with antioxidant and other health-promoting properties in fermented foods. Furthermore, microbial bioconversion during soybean fermentation may alter the profile and bioavailability of phenolic compounds, potentially influencing the functional properties of the resulting extracts. However, the present study focused primarily on polysaccharide-rich extracts and did not include metabolomic analyses of fermentation-derived compounds. Therefore, the specific metabolites generated during kefir fermentation and their contributions to the observed ACE inhibitory, antioxidant, and neuron cell-protective activities remain unclear. Future studies employing targeted or untargeted metabolomic approaches are warranted to further characterize these fermentation-derived metabolites and clarify their potential functional roles. Overall, the results indicate that oxidative degradation differentially influences the biological activities of soybean-derived kefir polysaccharides, enhancing antioxidant and ACE inhibitory activities while being associated with reduced protective effects in neuronal cell models. These findings provide valuable insights for tailoring the functional properties of soybean-derived kefir polysaccharides for targeted food and nutraceutical applications.

## 4. Conclusions

This study demonstrated that fermentation combined with oxidative degradation effectively enhanced the biofunctional properties of soybean-derived kefir polysaccharide-rich extracts. Oxidative degradation significantly improved antioxidant and ACE inhibitory activities, indicating that compositional modification plays an important role in regulating the biological activities of kefir-derived polysaccharides. Black soybean-derived extracts consistently exhibited stronger bioactivities than yellow soybean-derived extracts, highlighting the influence of raw material characteristics on functional performance. Furthermore, multivariate analysis revealed significant associations between compositional characteristics and bioactivities, providing valuable insights into the relationship between composition and functionality. Several limitations should be acknowledged. The biological activities were evaluated exclusively using in vitro assays, and the specific constituents responsible for the observed activities were not identified. Future studies should focus on the isolation and characterization of active polysaccharide fractions and peptide-derived components, together with mechanistic and in vivo investigations, to further validate their functional properties. Overall, these findings provide preliminary support for the potential of soybean-derived kefir polysaccharide-rich extracts as sources of bioactive constituents for food-related applications. However, further studies are required to validate these effects and determine their physiological relevance.

## Figures and Tables

**Table 1:** Molecular weight, chemical composition, and monosaccharide composition analyses for S-F, S-FD, B-F, and B-FD.

Molecular Weight	S-F	S-FD	B-F	B-FD
Molecular weight (kDa)	263.6	88.6	232.7	95.2
Chemical composition	S-F	S-FD	B-F	B-FD
Total sugar (%) *	29.3 ± 0.3 ^d^	10.5 ± 1.9 ^a^	22.5 ± 0.4 ^c^	13.8 ± 0.6 ^b^
Reducing sugar (%) *	0.50 ± 0.03 ^a^	8.67 ± 0.13 ^c^	0.80 ± 0.06 ^b^	11.84 ± 0.10 ^d^
Protein (%) *	2.47 ± 0.16 ^a^	3.16 ± 0.61 ^a^	5.12 ± 0.71 ^b^	5.35 ± 0.49 ^b^
TPC (%) *	0.53 ± 0.00 ^a^	2.06 ± 0.00 ^b^	0.46 ± 0.00 ^a^	2.10 ± 0.03 ^b^
TFC (%) *	0.006 ± 0.000 ^a^	0.065 ± 0.000 ^c^	0.039 ± 0.010 ^b^	0.065 ± 0.010 ^c^
Monosaccharide composition (molar ratio)	S-F	S-FD	B-F	B-FD
Mannose	1.000	1.000	1.000	1.000
Rhamnose	0.012	0.035	0.607	0.928
Glucuronic acid	0.144	0.113	0.004	0.021
Galacturonic acid	0.014	0.038	0.022	0.042
Glucose	0.007	0.003	0.001	0.019
Galactose	0.008	0.038	0.022	0.067
Xylose	0.050	0.133	0.038	0.005

* Values are mean ± SD (*n* = 3); TPC: total phenolic content; TFC: total flavonoid content; ^a–d^ data with different superscript letters in the same column are significantly different (*p* < 0.05).

**Table 2:** Composition of phenolic compounds for S-F, S-FD, B-F, and B-FD.

Phenolic Compounds (mg/g)	Samples			
	S-F	S-FD	B-F	B-FD
EGCG	71.1	0.9	0.3	82.7
Vanillic acid	0.3	4.0	1.2	97.4
Tannic acid	1.0	0.3	1.0	3.0
Catechin	-	-	-	1.4
Daidzein	0.3	-	-	0.7
Genistein	1.0	-	-	0.6
Gallic acid	-	0.2	0.2	0.1
Epicatechin	-	0.2	0.1	-
Homogentisic acid	-	1.5	1.1	-
Quercetin	0.2	-	-	0.1
ECG	-	0.1	0.1	-
Ferulic acid	0.1	-	-	0.1
Coumaric acid	0.1	-	-	0.1
Rutin	0.1	-	0.1	-
Chlorogenic acid	11.9	-	-	-
Mangiferin	0.1	-	-	0.1
CG	0.1	-	-	-
Ellagic acid	0.2	-	-	-

-: not detected. EGCG: epigallocatechin gallate; ECG: epicatechin gallate; CG: catechin gallate.

**Table 3:** Antioxidant activity analyses for S-F, S-FD, B-F, and B-FD.

Treatments	^A^ IC_50_ Value			12 mg/mL
	DPPH Radical Scavenging Activity (mg/mL)	ABTS^•+^ Scavenging Activity (mg/mL)	Ferrous-Ion Chelating Activity (mg/mL)	Reducing Power
				(OD 700 nm)
S-F	5.60 ± 0.10 ^d^	0.631 ± 0.023 ^d^	7.75 ± 0.12 ^d^	0.37 ± 0.00 ^a^
S-FD	1.12 ± 0.03 ^b^	0.0759 ± 0.0002 ^b^	1.32 ± 0.01 ^b^	1.40 ± 0.06 ^b^
B-F	3.43 ± 0.09 ^c^	0.237 ± 0.003 ^c^	3.83 ± 0.01 ^c^	0.34 ± 0.01 ^a^
B-FD	1.07 ± 0.02 ^b^	0.075 ± 0.0004 ^b^	1.24 ± 0.02 ^b^	1.68 ± 0.02 ^c^
BHA	0.01 ± 0.00 ^a^	0.0066 ± 0.0007 ^a^	-	3.00 ± 0.00 ^d^
EDTA	-	-	0.02 ± 0.00 ^a^	-

^A^ concentration of samples or standards required to scavenge 50% of 2,2-diphenyl-1-picrylhydrazyl (DPPH) radical scavenging, 2,2′-azino-bis(3-ethylbenzothiazoline-6-sulfonic acid) (ABTS) scavenging, or ferrous-ion chelating activities. Values are mean ± SD (*n* = 3); ^a–d^ data with different superscript letters in the same column are significantly different (*p* < 0.05). -: not detected.

## Data Availability

The original contributions presented in the study are included in the article; further inquiries can be directed to the corresponding authors.

## Abbreviations

The following abbreviations are used in this manuscript:

2-APB	2-aminoethoxydiphenyl borate
ABTS	2,2′-azino-bis(3-ethylbenzothiazoline-6-sulfonic acid)
ACE	angiotensin converting enzyme
BHA	butylated hydroxyanisole
BSA	bovine serum albumin
CG	catechin gallate
DNS	3,5-dinitrosalicylic acid
DPPH	2,2-diphenyl-1-picrylhydrazyl
ECG	epicatechin gallate
EDTA	ethylenediaminetetraacetic acid
EGCG	epigallocatechin gallate
FTIR	Fourier transform infrared
HPLC	high-performance liquid chromatography
NO	nitric oxide
PCA	principal component analysis
PI	propidium iodide
RNS	reactive nitrogen species
ROS	reactive oxygen species
ROT	rotenone
SEC	size exclusion chromatography
TFA	trifluoroacetic acid
TFC	total flavonoid content
TPC	total phenolic content
